# Frequency Function in Atomic Force Microscopy Applied to a Liquid Environment

**DOI:** 10.3390/s140609369

**Published:** 2014-05-26

**Authors:** Po-Jen Shih

**Affiliations:** Department of Civil and Environmental Engineering, National University of Kaohsiung, CEE NUK, No. 700, Kaohsiung University Rd., Nanzih District, 81148, Kaohsiung, Taiwan

**Keywords:** frequency shift function, jump to contact, liquid environment, atomic force microscopy

## Abstract

Scanning specimens in liquids using commercial atomic force microscopy (AFM) is very time-consuming due to the necessary try-and-error iteration for determining appropriate triggering frequencies and probes. In addition, the iteration easily contaminates the AFM tip and damages the samples, which consumes probes. One reason for this could be inaccuracy in the resonant frequency in the feedback system setup. This paper proposes a frequency function which varies with the tip-sample separation, and it helps to improve the frequency shift in the current feedback system of commercial AFMs. The frequency function is a closed-form equation, which allows for easy calculation, as confirmed by experimental data. It comprises three physical effects: the quasi-static equilibrium condition, the atomic forces gradient effect, and hydrodynamic load effect. While each of these has previously been developed in separate studies, this is the first time their combination has been used to represent the complete frequency phenomenon. To avoid “jump to contact” issues, experiments often use probes with relatively stiffer cantilevers, which inevitably reduce the force sensitivity in sensing low atomic forces. The proposed frequency function can also predict jump to contact behavior and, thus, the probe sensitivity could be increased and soft probes could be widely used. Additionally, various tip height behaviors coupling with the atomic forces gradient and hydrodynamic effects are discussed in the context of carbon nanotube probes.

## 1. Introduction

In the last ten years, the atomic-scale resolution of frequency-modulation atomic force microscopy (FM AFM) has been taken advantage of to image and conduct force spectroscopy measurements of biological samples in liquids. This has enabled quantitative measurements of conservative and dissipative forces, and subnanometer resolution imaging with piconewton-order loading forces at the solid and liquid interface. The thermal noise in the liquid environment increases the vertical vibration amplitude of the probe, hindering the FM AFM techniques from adopting probes with stiffness greater than 40 N/m [1]. The stiff probe is sensitive enough for strong interactions; however, weak interactions require a soft probe. Soft probes are highly susceptible to the problem of “jump to contact,” which is minor reason why the soft probe has not been adopted in FM AFM. On the other hand, Sader *et al.* [2] successfully overcame the amplitude problem of soft probes and constrained the vibration amplitude due to thermal noise under 2 nm for a probe stiffness of 3 N/m. This achievement indicates that the thermal noise problem of soft probes is solvable. To overcome the instability of “jump to contact”, this paper provides a function which can calculate frequency shifts, thus predicting “jump to contact” occurrences. The proposed frequency function could also be used in the tapping-modulation (TM) AFM and other forms of AFM. This frequency function is important for feedback control of AFM, and it can be used for the inverse calculation of tip-sample interaction forces in liquids.

FM AFM is a promising technique to measure biological samples. Recently, Bruker [3] developed a new TM AFM technique, referred to as Peak Force Quantitative Nanomechanical Mapping (PF QNM), applied to measure living and soft samples. PF QNM, succeeding from TM AFM, replaces detecting frequency (or phases) shifts with the peak force as the measurement feedback. Different from typical AFMs driven around resonance frequencies (around a hundred kilohertz), PF QNM is driven at a frequency far below (a few kilohertz). The probe amplitude of PF QNM is around hundred nanometers like general TM AFM, but that of FM AFM is around few nanometers. PF QNM features in measuring wide range of Young's moduli of biological samples with noise lower than general TM AFM. However, FM AFM, because of its small tip amplitudes of tip, has subnanometer resolution for measuring biological samples [1].

A probe selected in biological AFM depends on modulations of AFM and the resolution required for the specimen. Most atomic force microscopy surface imaging of bio-specimens is performed by the contact-modulated approach. To avoid destruction of the biological specimen [4], probe stiffness remains within the low range, 0.03–0.5 N/m [5,6]. However, low-stiffness probes easily jump to contact and suffer impaired resolution. For this reason, interest in the use of noncontact AFM has grown. FM AFM, one type of noncontact technique, uses low amplitude and measures the frequency shift of the probe, enabling higher resolution than other techniques. Within a liquid environment, atomic resolution can be achieved with biological applications of FM AFM, achieving a maximum signal by initial values of amplitudes at 0.6∼1.0 nm and leading a resonance frequency shift of tens of hertz [1]. Thermal noise makes control of the amplitude in the liquid difficult; the peak value of the power spectrum density of the thermal vibration is given by ${\left(z_{th}\right)}_{\text{peak}}=\sqrt{2{\text{k}}_{\text{B}}\text{TQ}/\pi {\text{f}}_{0}\text{k}}$, where *f*_0_ and *Q* are resonance frequency and the *Q* factor of the microcantilever, *k_B_* and *T* are Boltzmann's constant and absolute temperature, and *k* is the probe stiffness. Typically, we increase stiffness (the resonance frequency) to decrease the thermal amplitude of probe, thus, avoiding “jump to contact” issues. However, increasing stiffness decreases the sensitivity when measuring the atomic force. Reversing the assumption on increasing stiffness, we try to reduce the value of *Q* (for example, by improving the microcantilever shape) and consider the benefit of “jump to contact.” It may be possible to develop a highly sensitive probe in liquid. Thus, this paper adapts the use of soft probes and provides a frequency shift function which predicts “jump to contact” behavior.

The frequency function setup in commercial AFM devices can be improved easily. Scanning specimens in liquids using commercial atomic force microscopy (AFM) is time-consuming due the need for a try-and-error iteration to determine appropriate triggering frequencies. In addition, this iteration easily contaminates the AFM tip and damages samples, which consumes probes. One significant reason for this is that the frequency function is not accurate. The frequency function is not a function of the separation between tip and specimen. This results in the TM AFM incorrectly using resonant frequency to trigger the microcantilever and recording the wrong signals to judge height of specimen when the tip approaches the sample. The resonance frequency of AFM probes in liquid has been commonly approximated by *f* = *f_vac_* (1 + π*ρ_f_b*^2^/4*m*)^−1/2^, where *f_vac_* is the resonance frequency in vacuum, *ρ_f_* and *m* are the fluid density and mass per unit length, and *b* is the width of the probe. This approximation is suitable for the probe in far field, but it does not adequately represent the resonance frequency when the probe approaches close to the specimen. Unfortunately, this equation is used in commercial AFM devices, and the differences could be on the order of a few hundred hertz. Two causes underlie the shift in resonance frequency in liquids: (1) the interaction of atomic forces between the tip and the specimen, and (2) the disturbance of liquid in the space between the probe and the boundary surface on which the specimen is placed. In the first, atomic forces manifest over a few nanometers, and the effects to the shift can again be divided into two types: (a) the probe's eigenfunction varying with the tip–sample separation (the quasi-static equilibrium condition [7]), and (b) the force gradient effect around the amplitude region (Giessibl's theorem [8]). The effect of the disturbance of liquid is mainly based on Green and Sader's theorem [9] and arises from the liquid pressure on the probe varying with probe-surface separation. This effect manifests over tens of micrometers. Sader *et al.* suggested a frequency equation that considers the atomic force effect and the liquid pressure effect [1]. The aforementioned frequency function contains an opened-integration ( $\int_{z}^{\infty }$), which is ultimately problematic for AFM engineers. To improve it and allow for the use of soft probes, this paper provides an equation for the frequency shift as a function of tip-sample separation. The frequency function is a closed-from equation and is quick to obtain results. Experimental data are also proven with the proposed frequency function. Additionally, various tip height behaviors coupling with the atomic forces gradient and hydrodynamic effects are discussed in the context of carbon nanotube probes.

## 2. Mathematic Model and Discussion

### 2.1. Quasi-Static Equilibrium Condition

The equation of motion from the Euler-Bernoulli beam is:

(1) $$\frac{\partial^{2}}{\partial x^{2}}[EI\left(x\right)\frac{\partial^{2}w\left(x,t\right)}{\partial x^{2}}]+m\left(x\right)\frac{\partial^{2}w\left(x,t\right)}{\partial t^{2}}+c\left(x\right)\frac{\partial w\left(x,t\right)}{\partial t}=p\left(x,t\right)$$

**Figure 1.**
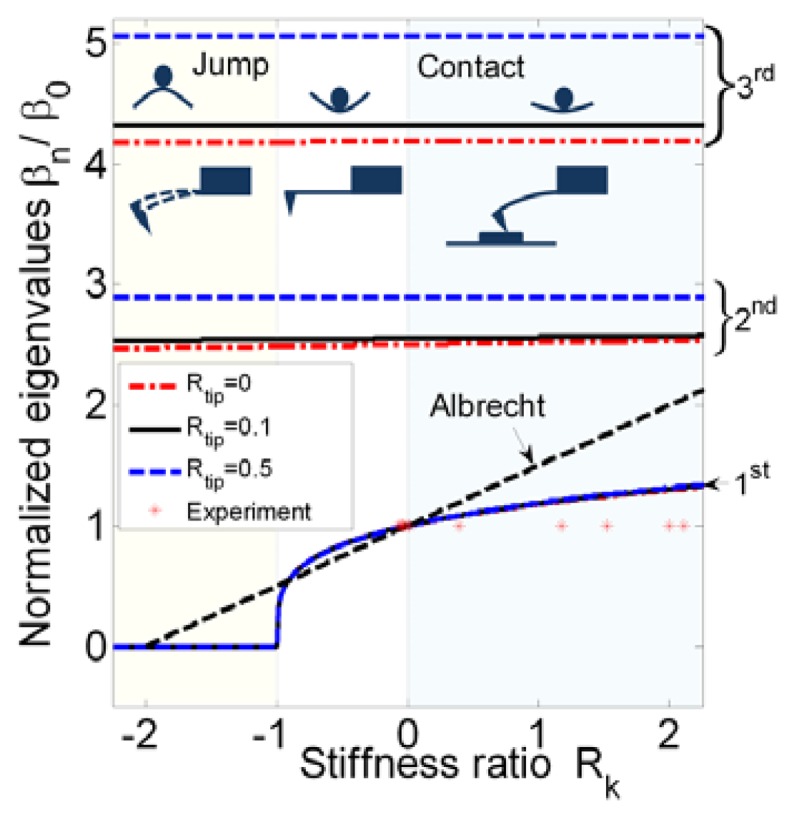
The first three normalized eigenvalues *β_n_*/*β*_0_ varying with stiffness ratio *R_k_* and tip-mass ratio *R_tip_*. *R_k_* > 0 represents a contact, −1 < *R_k_* < 0 represents a oscillation, and *R_k_* < −1 represents a jump. Experimental data, denoted by *, are from Gotsmann *et al.* [11].

The following discussion is limited to beams with uniform properties along their lengths, *i.e.*, Young's modulus, moment of inertia, mass per unit length, and the damping coefficient given by the constants: *E*, *I*, *m*, and *c*. The total displacement is *w*(*x*,*t*) as the sum of displacements that would be induced by static application of the support motion, *i.e.*, the quasi-static displacement, plus the additional displacement due to the dynamic inertial and viscous force effects [10]. At the clamped-end, *w*(0,*t*) = 0 and *w′*(0,*t*) = 0. At the tip-end, affected by the atomic force, *w″*(0,*t*) = 0 and *EIw‴*(*L*,*t*) = *m_tip_*ẅ(*L*,*t*) + *k_ts_*(*z*)*w*(*L*,*t*), in which *m_tip_* is the mass of the tip, *k_ts_*(*z*) is the gradient of the force curve, and *L* is beam length. Applying the above boundary conditions to Equation (1) gives the eigen function:

(2) $$\left(3R_{k}-R_{\text{tip}}\beta_{n}^{4}\right)\left(\sin\beta_{n}\cosh\beta_{n}-\cos\beta_{n}\sinh\beta_{n}\right)+\beta_{n}^{3}\left(1+\cos\beta_{n}\cosh\beta_{n}\right)=0$$

where *β_n_* is a coefficient in the separation of variable method, and the value is relative to the natural frequency of the microcantilever. The stiffness ratio and the tip-mass ratio are *R_k_* = −*k_ts_*/*k*_0_ and *R_tip_* = *m_tip_*/*mL*. *k*_0_ = 3*EI*/*L*^3^ is the stiffness of the microcantilever. If *k_ts_*(*z*) is assumed to be specific form (for example, Lennard-Jones form), *β_n_* can be solved by numerical method. The eigenfrequency is defined by $f_{\text{n}}\left(R_{k},R_{up}\right)=\sqrt{\frac{\text{EI}}{\text{m}}}{\left(\beta_{\text{n}}/\text{L}\right)}^{2}/2\pi$. The first three modes of the eigenvalues *β*_1_, *β*_2_, and *β*_3_ *versus* the stiffness ratio for certain tip-mass ratios are plotted in Figure 1. All eigenvalues are normalized by *β*_0_, the eigenvalue when *R_k_* = 0. Thus *β*_0_ leads to *f*_0_, which represents the resonance frequency in the far field. From above, the resonance frequency is not constant, as it varies with tip-sample separation. Experimental data obtained from Gotsmann *et al.* is also plotted [11]. The minimum stiffness ratios in the associated experiments are shown to be a little bit less than zero (*R_k_* → 0^−^).

However, the first eigenvalue may sometimes have a trivial solution when *R_k_* ≤ −1. A critical point, (*R_k_*, *β*_1_) = (−1,0) is utilized to determine where static equilibrium is broken and a jump occurs. This can be proven by applying an asymptotic series in *β*_1_. Here, substituting sin*β*_1_ = *β*_1_ − *β*_1_^3^/3! + ⋯, cos*β*_1_ = *β*_1_ − *β*_1_^2^/2! + ⋯, sin*hβ*_1_ = *β*_1_ + *β*_1_^3^/3! + ⋯, and cos*hβ*_1_ = *β*_1_ + *β*_1_^2^/2! + ⋯ into Equation (2) gives (3*R_k_* – *R_tip_β*_1_^4^)(2*β*_1_^3^/3) + 2*β*_1_^3^ = 0, in which the higher order terms are neglected. Setting *β*_1_ → 0 leads to *R_k_* = −1. This implies that the absolute value of the gradient of atomic force is the same as the stiffness of the beam. This corresponds to the case where the tip approaches the sample from afar, and the stiffness ratio decreases from 0 to −1. The minimum stiffness ratio of a stiff microcantilever may not reach −1, but a soft microcantilever may reach −1. Then a small disturbance brings the soft cantilever into the region *R_k_* < −1, where the attractive force exceeds the restoring force, accelerating the tip toward the sample. In Figure 1, this nonlinear result breaks the assumption that the frequency shift is a linear function of the tip–sample gradient in the range [−1, 0], Δ*f* = *f*_0_/2(*k_rs_*/*k*_0_) as provided [12]. Figure 1 also shows that the second and third eigenvalues remain constant with the stiffness ratio. It is also clear that the tip-mass ratio influences the eigenvalues, but is independent of the tip-sample separation. From an experimental data point of view, typical probes are conservatively designed according to (*R_k_* → 0^−^). Since the performance of the probe can be predicted in our proposal, the stiffness ratio range for an effective probe can be expanded to −1 < *R_k_* < 0.

### 2.2. Atomic Force Gradient Effect

Moreover, the frequency is a function of vibration amplitude, especially oscillation in the near field featuring rapid variation of the atomic force. Giessibl modified the results of Albrecht *et al.* [12] and utilized canonical perturbation theory to solve the frequency shift for large amplitudes [8]. His solution assumes that the gradient of the restoring force is larger than that of the atomic force, and that the cantilever motion is well described as approximately harmonic. Then the frequency shift is strictly proportional to *f_n_* and 1/*k*_0_, *i.e.*, Δ*f* = (*f_n_*/2*k*_0_)<k*(*A*_0_)>, in which the bracket, < >, indicates averaging across one oscillation cycle and is the weighted average of the gradient of the force:

(3) $$\langle k^{*}\left(A_{0}\right)\rangle =\frac{2}{\pi A_{0}^{2}}\int_{-A_{0}}^{A_{0}}k_{ts}\left(x\right)\sqrt{A_{0}^{2}-x^{2}}dx$$

Giessibl applied to the atomic resolution of his studies, but the equilibrium of the quasi-static conditions was not considered [13–15]. *f*_0_ remains constant in his papers. However, his experimental frequency shifts were still well matched with the theoretical prediction. Because the probes employed in these experiments were sufficiently stiff, *R_k_* → 0^−^, so *f*_0_ was constant. In this paper, the combination of eigen frequencies obtained from the quasi-static equilibrium and Giessibl's theory leads to the resonance frequency in vacuum:

(4) $$f_{\text{vac}}\left(R_{k},R_{\text{tip}},A_{0}\right)=f\left(R_{k},R_{\text{tip}}\right)[1+\frac{1}{\pi k_{0}A_{0}^{2}}\int_{-A_{0}}^{A_{0}}k_{ts}\left(z-x\right)\sqrt{A_{0}^{2}-x^{2}}dx]$$

**Figure 2.**
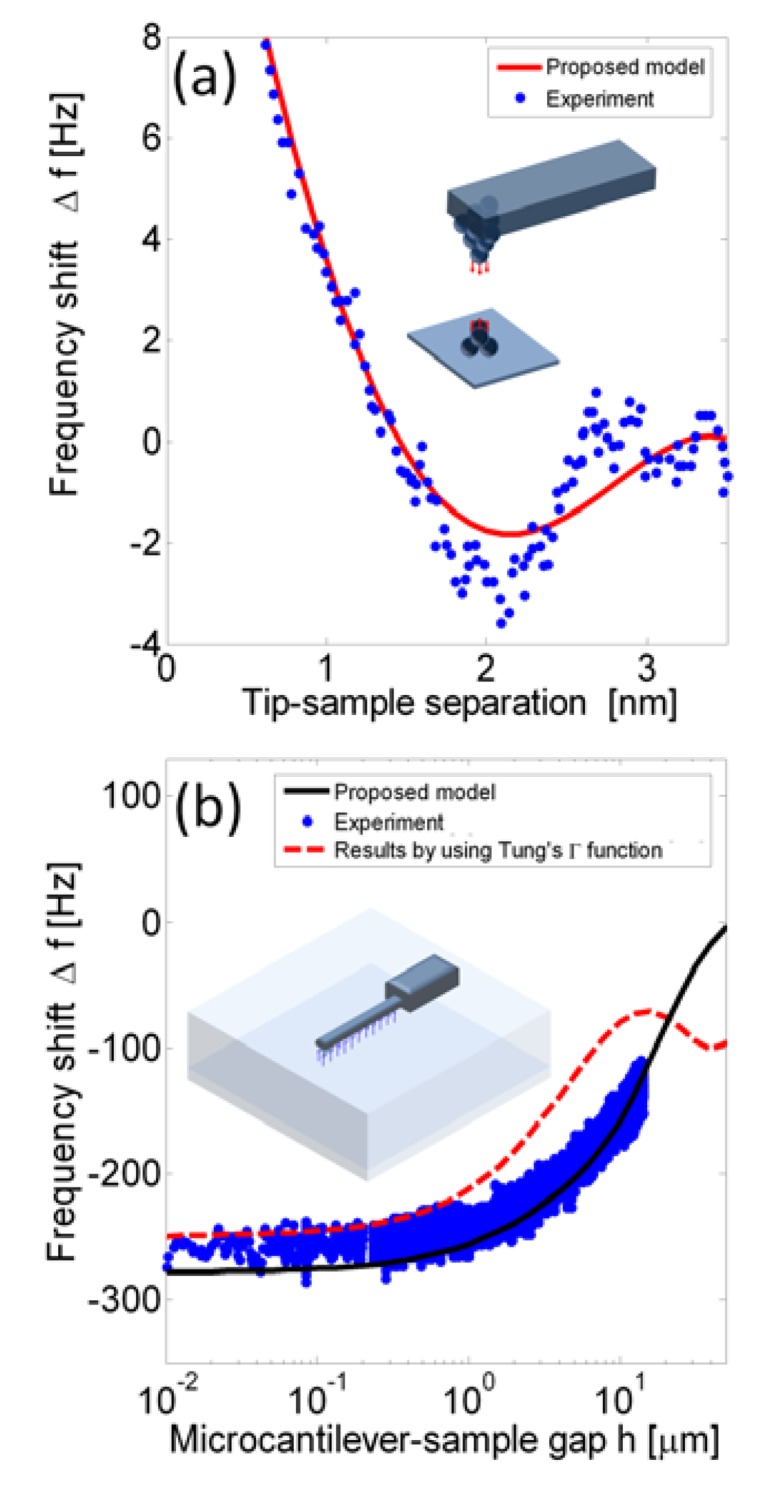
Frequency shift *versus* separation for: (**a**) a vacuum case with prediction by the proposed method (solid line) and experimental data (dots) [16], and (**b**) a liquid case with prediction by proposed method (solid line), experimental data (dots) [2], and results (dashed line) by the proposed method but with Γ*_r_* replaced by approximation [21].

If *k_rs_*(*z*) is assumed to be Lennard-Jones' formula, the integration could be solved to be a function of amplitude. Figure 2a shows the frequency shift of a tungsten tip on a KCl (100) surface in which Equation (4) is applied to compare with experimental data from Giessibl's study [16]. Model data was generated with an amplitude *A*_0_ = 0.15 nm, a stiffness of *k*_0_ = 1800 N/m, length 2.4 mm, width 130 μm, thickness 214 μm, and eigenfrequency set to *f*_1_ = 25,068 Hz.

### 2.3. Hydrodynamic Effect

The modeled frequency shift takes into account hydrodynamic effects when the probe is immersed in liquid. Sader [9] extended the boundary integral technique of Tuck [17] and presented an explicit semi-analytical theory to solve for an oscillating cantilever immersed in a viscous fluid nearby the specimen surface. This paper extended Sader's work to FM AFM probe. It assumes that the fluid was incompressible and the oscillation amplitude was small. The fluid flow around the microcantilever is governed by the incompressible unsteady Stokes equation:

(5) $$\rho_{f}\frac{\partial \text{v}}{\partial t}=-\nabla P+\eta \nabla^{2}\text{v}$$

where v, *P*, and *η* are vector velocity, pressure, and viscosity of the fluid, respectively; and the Reynolds number of the flow is *R_e_* = π*ρ_f_f_h_b*^2^/2*η* [18], and the Reynolds number of a soft AFM probe of width 30 μm oscillating in water is in range [0.1, 20]. Fluid velocities are restricted to be normal to the solid surface, whereas no-slip boundary conditions are enforced at interfaces. The hydrodynamic load per unit is obtained from pressure differences between the top and bottom of the beam, $p_{h}=\eta \dot{\text{w}}\left(\text{x},\text{t}\right)\int_{-\text{b}/2}^{\text{b}/2}\Delta P\left(\xi \right)\text{d}\xi$, in whichẇ(x, t) is the normal velocity and Δ*P*(*ξ*) is the pressure jump across the beam. Let the Fourier transform be $\tilde{\text{X}}=\int_{-\infty }^{\infty }{\text{Xe}}^{\text{iwt}}\text{dt}$. Taking the Fourier transform of Equation (1) yields EIw̃⁗ −mω^2^w̃+iωcw̃ = p̃. p̃ is the hydrodynamic loading component. Examining the Fourier transformed equations of Equation (5) and setting ∇· **v** = 0 give the general form of the hydrodynamic loading component as p˜=π3fn2ρfb2Γ(Re,b,h)w˜, where *h* is the gap between the probe and the surface and $\Gamma \left(R_{e},b,h\right)=i\int_{-\text{b}/2}^{\text{b}/2}\Delta P\left(\xi \right)\text{d}\xi /\left(\pi R_{e}\right)$ is the complex hydrodynamic function [19]. Its real part Γ*_r_* represents the effect of added mass on the surrounding fluid, and its imaginary part Γ*_i_* represents the damping effect of the fluid. The hydrodynamic load equation is formally exact in the limit of 0 < *L*/*b* ≪ 1. Substituting p̃ into the Fourier-transformed motion equation leads *f_h_*(*R_e_*,*b*,*h*) = *f_vac_* [1 + π*ρ_f_b*^2^Γ*_r_*(*R_e_*,*b*,*h*)/4*m*]^−1/2^. Note the other formula for the frequency function can refer to the study by Naik *et al.* [20]. Thus *f_vac_* replaced by Equation (4) gives the frequency:

(6) fh(Rk,Rtip,A0,Re,b,h)=fn(Rk,Rtip)1+1πk0A02∫−A0A0kts(z−x)A02−x2dx[1+πpfb24mΓr(Re,b,h)]1/2

This is the desired equation, representing frequency as a function of the eigenfrequency and proportional to the atomic effect and inversely proportional to the hydrodynamic effect. From an engineering point of view, the interaction force curve can be assumed and the probe properties and the amplitude of the probe are given by users. This frequency function works while the hydrodynamic function Γ*_r_* is given. Here, Γ*_r_* has an approximation function and will be discussed later. Note that Γ*_r_* is a function of the Reynolds number *R_e_*, which itself is a function of *f_h_*. Applying the numerical method allows *f_h_* to be easily solved. As a result, Equation (6) is useful in commercial devices. This equation is different to the equation provided by Sader *et al.* [1]. It contains the quasi-static equilibrium condition and can predict the performance of the soft AFM probes. Furthermore, the inverse method for calculating the tip-sample forces in liquid from the experimental frequency shifts can be realized by applying Giessibl's technique [16] along with Equation (6).

### 2.4. Hydrodynamic Function

The details of the hydrodynamic function Γ*_r_*(*R_e_*,*b*,*h*) can be obtained from the works of Green, Sader, and van Eysden [9,19] who provided closed-form analytical expressions for the entries of all submatrices except four complicated terms, (*A*_1_,*A*_2_,*C*_1_,*C*_3_). In this paper, these four terms were computed by applying numerical Gauss-Legendre quadrature. But it was a time consuming procedure. Tung *et al.* [21] calculated the hydrodynamic function and provided the polynomial approximation function by curve fitting in the domain *R_e_* ∈[10^−2^,10^4^]. These fitting results were found to have errors on the order of a few percent, which might shift the frequency a few tens of hertz (the eigenfrequency is around one hundred kilohertz).

Figure 2b shows the frequency shift of a microcantilever immersed in water plotted against the gap between the probe and the surface. The dimensions of the microcantilever (Nanosensors EFM cantilever) are length 225 μm, width 23 μm, thickness 3 μm, a tip height of 13 μm, a oscillation amplitude of 1 nm, and the resonance frequency in water is 13.14 kHz. The solid line plots results obtained from Equation (6) directly by the semi-analytical approach [9]. The dashed line represents results from Equation (6) but with the hydrodynamic function derived from Tung's polynomial approximation [21]. The dots are experimental data obtained from Sader *et al.* [2]. The dashed line deviates when the gap is larger than 10 μm because Tung's approximation is outside the convergent region. Tung's approximation saves time and is useful in predicting the shift tendency of the resonance frequency. When the gap is much smaller than the microcantilever width, a change in the gap has less of an effect on the resonance frequency due to the hydrodynamic effect. Comparing Figure 2a with 2b, the atomic force affects the frequency shift by several hertz in a region a few nanometers in the near-field, and the hydrodynamic affects the frequency shift by hundreds of hertz in the region around a hundred micrometers. As a result, while the tip height is a few micrometers, these two effects are coupled.

### 2.5. Tip Heights and Frequency Shifts

**Figure 3.**
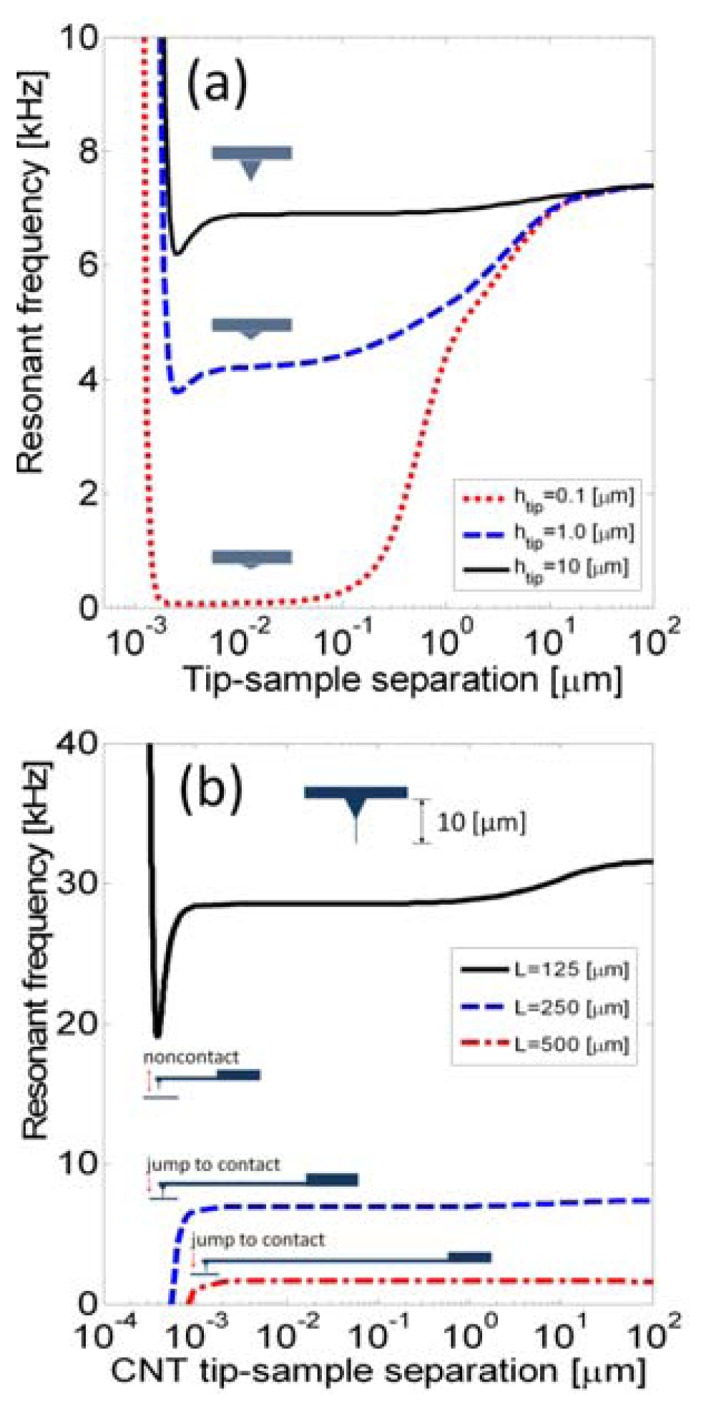
Frequency *versus* tip-sample separation for (**a**) probes with various tip heights, and (**b**) a CNT-tip with various microcantilever lengths.

Figure 3a shows frequency shift *versus* tip-sample separation for various tip heights, set at 0.1, 1, and 10 μm. A case of a silicon tip and a polystyrene surface is studied with probe dimensions of length 250 μm, width 35 μm, and thickness 5.7 μm.

The resonance frequency in water is 232.5 kHz. The Lennard-Jones interaction force is assumed to be *F*(*z*) = *A*_1_*R*/180*z*^8^ − *A*_2_*R*/6*z*^2^, in which *R* = 20 nm, *A*_1_ and *A*_2_ are the Hamaker constants, 0.838873 × 10^−70^ J m^6^ and 1.15072 × 10^−19^ J, respectively [22]. The results indicate that the *h*_tip_ = 0.1 μm case presents the hydrodynamic affects from far field to the near field, tip-sample separation between 0.1−10 μm. Hydrodynamic effects are effectively constant when the tip-sample separation is less than 0.1 μm. Moreover, typical tips are around ten micrometers, and the frequency shifts due to the hydrodynamic force are on the order of hundreds of hertz, as shown *h*_tip_ = 10 μm. Hence, high tips feature reducing of the hydrodynamic and presenting the atomic force effects.

Recently, tips extended by a carbon nanotube (CNT) positioned on the tip have been used to measure high-aspect ratio samples. The CNT can be hundreds of nanometers to a few micrometers long, and a few hundred nanometers long is suggested to avoid buckling. In the following calculation, the total height of a CNT-tip is set to 10 μm, and the rest of the probe geometry is the same as in previous calculations. The carbon-carbon interaction potential is *F*(*z*) = 4*επnσ*^2^[−(*σ*/*z*)^10^/5+ (*σ*/*z*)^4^/2], where the parameters are assumed to be *ε* = 4.751×10^−22^ eV, *n* = 0.114 Å^−3^, and *σ* = 3.407 Å [23]. To study the jumps, the microcantilever length was set sequentially to *L* = 125, 250, and 500 μm (*k*_0_ distribution ratio is 1/8:1:8) as shown in Figure 3b. Since the CNT-tip has a rapid interaction, the stiffness ratio becomes less than −1 in the first two cases, and that represents CNT-tip jumps. The frequency shifts and critical points of the jumps can also be analytically obtained by the proposed method.

## 3. Conclusions

A frequency function for AFM in liquid has been introduced, with the frequency shift derived as a function of six parameters: stiffness ratio, mass ratio, oscillation amplitude, Reynolds number, probe width, and tip height. This proposed frequency function is in closed form, which is easy for engineers to implement in AFM devices. It can predict the jump to contact behavior. Accordingly, probes with stiffness ratios smaller than −1 could be applied; that is soft probes could be easily used to increase the probe sensitivity. By application of the frequency function, the probe with high tips demonstrates the effect of coupling between atomic and hydrodynamic forces, and soft probes with CNT-tips show jumping behavior in accord with the predictions.

## References

[1] Fukuma T., Jarvis S.P., Morita S., Giessibl F.J., Wiesendanger R. (2009). Biological applications of FM-AFM in liquid environment. Noncontact Atomic Force Microscopy.

[2] Sader J.E., Uchihashi T., Higgins M.J., Farrell A., Nakayama Y., Jarvis S.P. (2005). Quantitative force measurements using frequency modulation atomic force microscopy—Theoretical foundations. Nanotechnology.

[3] Adamcik J., Berquand A., Mezzenga R. (2011). Single-step direct measurement of amyloid fibrils stiffness by peak force quantitative nanomechanical atomic force microscopy. Appl. Phys. Lett..

[4] Xu X., Carrasco C., de Pablo P.J., Gomez-Herrero J., Raman A. (2008). Unmasking imaging forces on soft biological samples in liquids when using dynamic atomic force microscopy: A case study on viral capsids. Biophys. J..

[5] Melcher J., Carrasco C., Xu X., Carrascosa J.L., Gomez-Herrero J., Jose de Pablo P., Raman A. (2009). Origins of phase contrast in the atomic force microscope in liquids. PNAS.

[6] Kodera N., Yamamoto D., Ishikawa R., Ando T. (2010). Video imaging of walking myosin V by high-speed atomic force microscopy. Nature.

[7] Shih P.J. (2012). Tip-jump response of an amplitude-modulated atomic force microscope. Sensors.

[8] Giessibl F.J. (1997). Forces and frequency shifts in atomic-resolution dynamic-force microscopy. Phys. Rev. B.

[9] Green C.P., Sader J.E. (2005). Small amplitude oscillations of a thin beam immersed in a viscous fluid near a solid surface. Phys. Fluids.

[10] Clough R.W., Penzien J. (2003). Dynamics of Structures.

[11] Gotsmann B., Anczykowski B., Seidel C., Fuchs H. (1999). Determination of tip-sample interaction forces from measured dynamic force spectroscopy curves. Appl. Surf. Sci..

[12] Albrecht T.R., Gütter P., Horne D., Rugar D. (1991). Frequency modulation detection using high-Q cantilevers for enhanced force microscope sensitivity. J. Appl. Phys..

[13] Giessibl F.J. (1995). Atomic resolution of the silicon (111)-(7 × 7) surface by atomic force microscopy. Science.

[14] Hembacher S., Giessibl F.J., Mannhart J. (2004). Force microscopy with light-atom probes. Science.

[15] Welker J., Giessibl F.J. (2012). Revealing the angular symmetry of chemical bonds by atomic force microscopy. Science.

[16] Giessibl F.J. (2001). A direct method to calculate tip-sample forces from frequency shifts in frequency-modulation atomic force microscopy. Appl. Phys. Lett..

[17] Tuck E.O. (1969). Calculation of unsteady flows due to small motions of cylinders in a viscous fluid. J. Eng. Math..

[18] Sader J.E. (1998). Frequency response of cantilever beams immersed in viscous fluids with applications to the atomic force microscope. J. Appl. Phys..

[19] Van Eysden C.A., Sader J.E. (2006). Resonant frequencies of a rectangular cantilever beam immersed in a fluid. J. Appl. Phys..

[20] Naik T., Longmire E.K., Mantell S.C. (2003). Dynamic response of a cantilever in liquid near a solid wall. Sens. Actuators A.

[21] Tung R.C., Jana A., Raman A. (2008). Hydrodynamic loading of microcantilevers oscillating near rigid walls. J. Appl. Phys..

[22] Rützel S., Lee S.I., Raman A. (2003). Nonlinear dynamics of atomic-force-microscope probes driven in Lennard-Jones potentials. Proc. R. Soc. Lond. A.

[23] Kutana A., Giapis K.P., Chen J.Y., Collier C.P. (2006). Amplitude response of single-wall carbon nanotube probes during tapping mode atomic force microscopy: Modeling and experiment. Nano Lett..

